# How the latent geometry of a biological network provides information on its dynamics: the case of the gene network of chronic myeloid leukaemia

**DOI:** 10.3389/fcell.2023.1235116

**Published:** 2023-11-24

**Authors:** Paola Lecca, Giulia Lombardi, Roberta Valeria Latorre, Claudio Sorio

**Affiliations:** ^1^ Faculty of Engineering, Free University of Bozen-Bolzano, Bolzano, Italy; ^2^ Department of Mathematics, University of Trento, Trento, Italy; ^3^ General Pathology Division, Department of Medicine, University of Verona, Verona, Italy

**Keywords:** network geometry, graph embedding, dynamical systems, spring systems, chronic myeloid leukaemia, systems biology

## Abstract

**Background:** The concept of the latent geometry of a network that can be represented as a graph has emerged from the classrooms of mathematicians and theoretical physicists to become an indispensable tool for determining the structural and dynamic properties of the network in many application areas, including contact networks, social networks, and especially biological networks. It is precisely latent geometry that we discuss in this article to show how the geometry of the metric space of the graph representing the network can influence its dynamics.

**Methods:** We considered the transcriptome network of the Chronic Myeloid Laeukemia K562 cells. We modelled the gene network as a system of springs using a generalization of the Hooke’s law to *n*-dimension (*n* ≥ 1). We embedded the network, described by the matrix of spring’s stiffnesses, in Euclidean, hyperbolic, and spherical metric spaces to determine which one of these metric spaces best approximates the network’s latent geometry. We found that the gene network has hyperbolic latent geometry, and, based on this result, we proceeded to cluster the nodes according to their radial coordinate, that in this geometry represents the node popularity.

**Results:** Clustering according to radial coordinate in a hyperbolic metric space when the input to network embedding procedure is the matrix of the stiffnesses of the spring representing the edges, allowed to identify the most popular genes that are also centres of effective spreading and passage of information through the entire network and can therefore be considered the drivers of its dynamics.

**Conclusion:** The correct identification of the latent geometry of the network leads to experimentally confirmed clusters of genes drivers of the dynamics, and, because of this, it is a trustable mean to unveil important information on the dynamics of the network. Not considering the latent metric space of the network, or the assumption of a Euclidean space when this metric structure is not proven to be relevant to the network, especially for complex networks with hierarchical or modularised structure can lead to unreliable network analysis results.

## 1 Introduction

With the emergence of systems biology around the year 2000, the representation of a system of interacting biological entities, such as proteins, molecules, functional complexes, *etc.*, in the form of a network or graph has become preponderant and an unreliable prerequisite of any mathematical model regarding both the static and dynamic properties of the network. This representation of the components of a system as network nodes and their interactions as arcs between the nodes proved to be easy to understand as it is intuitive and also an excellent tool for organising data. However, the immediacy of understanding such a representation comes at the price of its low informational power, its susceptibility to misinterpretation and its use that often takes place under tacit or even unconscious assumptions. Particularly in the graph representation of a network, it is natural to think of the concept of distance between nodes as the number of arcs separating the nodes, or, if the weights of the arcs are known, as the weighted sum of the number of arcs separating the nodes. In doing so, it is implicitly assumed that the distance between two nodes is a Euclidean distance, or, in other words, that the metric space in which the network resides is flat Euclid space. This implicit assumption on a measure as important as the distance between nodes, used in multiple contexts as a measure of the intensity of an interaction between nodes, if not of the propensity of the interaction itself, may not only be reductive or approximate, but may even be incorrect. An erroneous assumption about the metric space that represents the geometry of the network carries serious risks, one of which is that of not being able to grasp the organizational principles of the typology and consequently the dynamics of the network. Indeed, the distribution of widely used centrality metrics like as degree and clustering coefficient reflect the features of the metric space, which defines the network’s geometry. For example, heterogeneous degree distributions and significant clustering emerge naturally as reflections of the underlying hyperbolic geometry’s negative curvature and hyperbolic metric characteristic (Krioukov et al., 2010). On the opposite, if a network has some metric structure and a heterogeneous degree distribution, the network has an effective hyperbolic geometry below (Krioukov et al., 2010).

It is often said to indicate the metric space of a network that the graph representing the network is “embedded” in a metric space, which is called the latent geometry of the network. The adjective “latent” is justified by the fact that the graph representation of a network does not make visible the characteristics of the metric space in which the coordinates of the nodes are actually defined. The verb “to embed”, on the other hand, although commonly used, we condemn somewhat misleadingly, since the network, if endowed with a metric structure, is in fact not embedded in a metric space as if it were a distinct entity that fits into it, but is itself a portion of it, more precisely a discrete version of the continuous metric space that represents it. The use of the verb “to embed” stems from the procedures dedicated to understanding what the latent geometry of the network might be and based on tests in which the network is considered to have metrics of a different nature and then the distortion that the new metric has with respect to the original metric defined by the network’s similarity matrix (i.e., weighted adjacency matrix) is assessed.

The latent geometry of a network is an important area of study in network science. We refer the reader to Boguñá et al. (2021); Jhun (2022) or an overview of the studies and fields of application of the study of the latent geometry of a network. In Jhun (2022), it is reported that latent geometry has been used to travel networks efficiently (Kleinberg, 2000; Boguñá et al., 2010) detect missing links (Liben-Nowell and Kleinberg, 2007; Clauset et al., 2008), map the brain (Allard and Serrano, 2020), and analyse proximity network (Papadopoulos and Flores, 2019). Interestingly, it has been shown that the map of contagions of various pandemics develops through paths defined on the latent geometry of the network of contacts and movements of individuals (Taylor et al., 2015). Systems biology has also benefited from the results of latent network geometry analysis, in particular the study of genetic networks and protein-protein interaction networks as reported in (Alanis-Lobato et al., 2018; Härtner et al., 2018; Pio et al., 2019; Klimovskaia et al., 2020; Sun et al., 2021; Lecca and Re, 2022; Lecca, 2023; Seyboldt et al., 2022).

While we can say that the relationship between latent geometry and static topological properties of the network, such as those measured by the centrality indices, is well established, the relationship between latent geometry and network dynamical properties is little investigated. A recent attempt in this direction was made by Rand et al. (2021). In this paper, Rand et al. study embryonic development. From egg to adult, embryonic development results in the reproducible and organised manifestation of complexity. In this process, the activity of gene networks culminates in the sequential differentiation of distinct cell types that construct this complexity, which has been likened by Conrad Waddington metaphor (Fard et al., 2016; Squier, 2017; Sánchez-Romero and Casadesús, 2021) to a flow through a landscape with valleys representing alternative destinies. Geometric approaches enable the formal description of such landscapes and codify the types of behaviours produced by differential equation systems.

With this study of ours, we wish to make a contribution in this still very unexplored field of the relations between latent geometry and the evolution of a network, particularly a biological network. We propose a method to infer the equations governing the dynamics of a network of genes previously identified by the authors (Lombardi et al., 2022) as involved in the development and progression of Chronic Myeloid Leukaemia (CML). The method consists of two steps: i) the determination of the latent geometry of the network through embedding of the network in three models of metric space (Euclidean, hyperbolic, and spherical), and ii) the determination of the dynamic equations describing this metric space. If the result of the step i) is the hyperbolic metric, the parameter of the dynamics of the interactions in the network conceived as a subspace of a hyperbolic space will depend on the hyperbolic distance between the interacting partners. Similarly, if the result of step i) is a spherical metric, the dynamics of the network will be parametrized by distance of the interacting nodes in the spherical space, and finally, if the result of step i) is an Euclidean metric, the network will be a dynamical systems whose parameters will depend on Euclidean distance between the interacting nodes.

In this study, we conceive of a network as a system of springs, in which the nodes constitute the masses and the arcs the springs that connect these masses/nodes. The spring constant represents the transmission efficiency of the interaction between the nodes. The interaction between a node A and a node B is seen as a change in the state of A causing a change in B. In accordance with the spring model, the interaction between nodes is seen as a propagation of the alteration of A’s state through the spring to B, which absorbs the alteration of A in turn changing its state. The vibrational states of the networks nodes are governed by a generalization of the Hook law. According to this law, the spring constant is calculated by dividing the force required to stretch or compress a spring by the lengthening or shortening of the spring. It is stated mathematically as *k* = −*F*/Δ*x*, where Δ*x* is the displacement of the mass, *F* is the force applied over *x*, and *k* is the spring constant (also known as *spring stiffness*). The propagation velocity of the elastic wave in a spring stressed by a force is directly proportional to the square root of the spring’s elastic constant. A stiffer spring has a greater spring stiffness, and *vice versa*. As a consequence, a high spring stiffness is interpreted as high efficiency and thus greater ease in the transmission of interaction between nodes. The elastic constant metaphor, in network metric space, corresponds to a measure of similarity between nodes, such that nodes connected by harder springs are closer nodes in terms of similarity. In the model of network we present here, the elastic constants of the springs are obtained from a generalization of the Hook’s law for a system with *N* masses and *E* springs (N corresponding to the number of nodes and E corresponding to the number of edges), where the mass of the node is given by its total degree and the change in the position of the node is given by the index of vibrational centrality proposed by Estrada and Hatano (2010). The matrix of elastic constants is used in network embedding procedures in three types of space, Euclidean, hyperbolic, and spherical. The metric space for which the embedding of the network shows a minimum distortion of the values of this matrix is considered as the best approximation for the metric space of the network. The distances of the nodes in this metric space constitute the parameters of the network dynamics, which we describe here in terms of mass action law.

**FIGURE 1:**
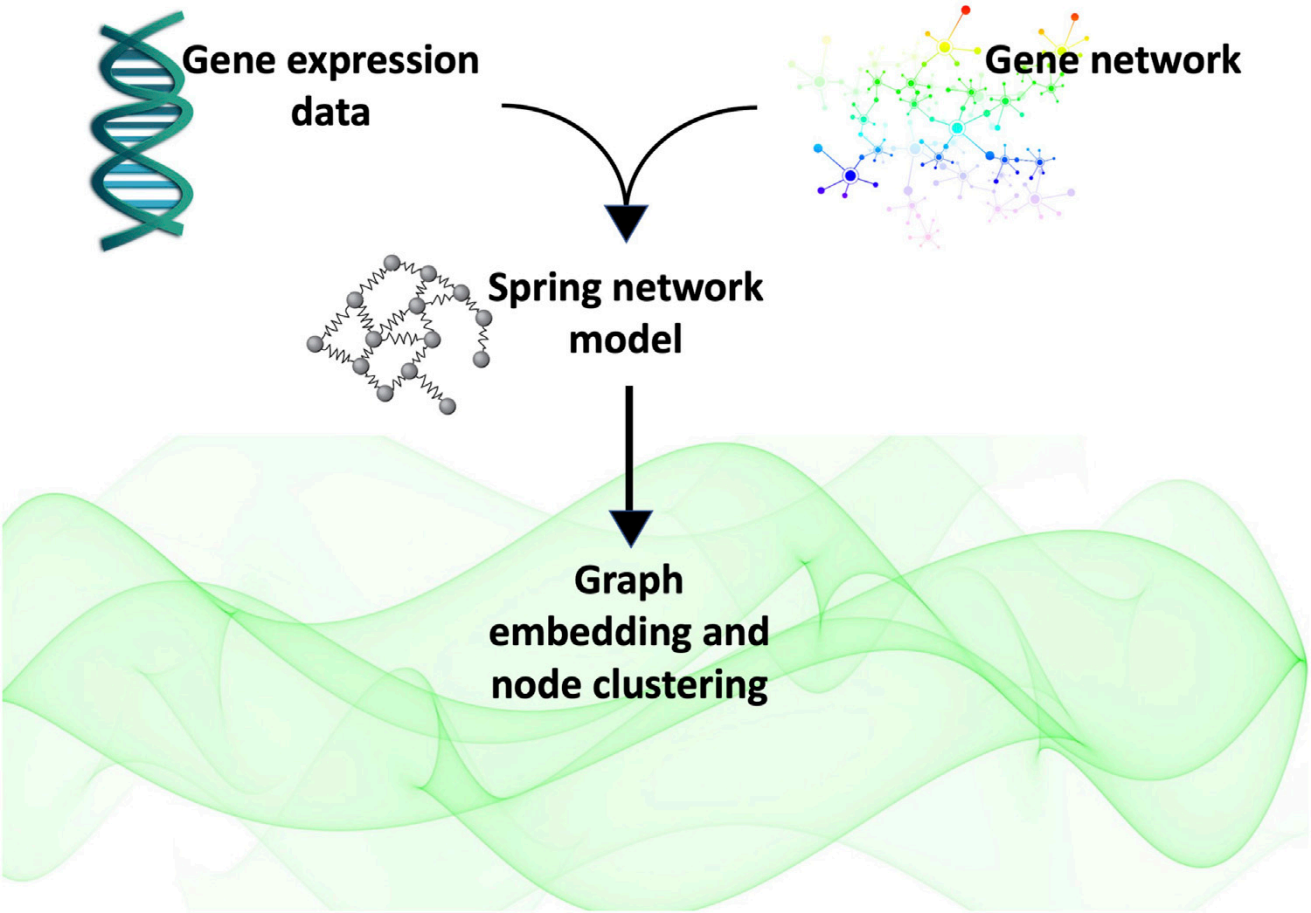
In this study, we obtained the gene network of interest by querying the Pathway Commons database with the list of genes of interest. We represented the network as a system of springs whose masses are the expression level of the genes as measured in our experiments in (Lombardi et al., 2022). We calculated the weighted adjacency matrix of the network as that matrix whose entries are given by the spring constant calculated at equilibrium. Finally, we used this matrix to embed the graph into three spaces (flat, positively curved and negatively curved)space) in order to determine which of them best represented the network’s latent geometry. Finding the hyperbolic space fits best the latent geometry of the network, we proceeded to cluster the nodes according to their radial coordinate, that representative of the node popularity (Papadopoulos et al., 2012).

The article is organised as follows: in Section 2 we introduce the three types of isotropic spaces considered in this study and the embedding techniques we used to identify which of the metric spaces considered best represents the network’s latent geometry. In Section 3 we describe the data and the gene network of the case study. In Section 4, we describe the mathematical modelling of the gene network as a system of springs, and finally in Section 5 we report the results obtained. This is followed by some concluding remarks and a recapitulation of the study performed (Section 6). In Figure 1 we illustrate the main steps of the analysis presented in this study.

## 2 Network geometry and methods of embedding

A graph embedding consists in the determination of the coordinates of the graph node in a given metric space in such a way that the graph similarity matrix is reproduced with as little error as possible. The embedding of a graph thus consists of the problem of finding the coordinates of the nodes in a given metric space from the similarity matrix of the graph, which is a measure of the distances between nodes. In the final analysis, embedding consists of finding coordinates of points given their distance. Isotropic spaces can only be classified as Euclidean (flat), elliptic (having positive curved), or hyperbolic (having negative curvature). In the following sections, we will recall some basic definitions, such as that of inner product and distance for these three types of spaces, and briefly mention the mathematical techniques of graph embedding, of which there are many variants in literature. We also recall how the latent geometry is related to the structure and organizational principles of the network (e.g., presence of communities, hierarchical organization, etc.).

### 2.1 Euclidean space

The Euclidean geometry is based on the following five postulates: (i) Any two points can be joined by a straight line segment. (ii) Any portion of a straight line can be stretched forever. (iii) Any straight line segment can be used as the radius of a circle, with one endpoint serving as the centre. (iv) All right angles are congruent. (v) When two lines are drawn so that they intersect a third in a fashion that results in a side where the total of the inner angles is less than two right angles, the two lines will always cross each other if they are extended far enough.

More formally, an Euclidean space, is a real vector space (i.e., a vector space whose field of scalars is R ) *E* equipped with a positive definite symmetric bilinear form φ:E×E→R . The real number *φ* (*x*, *y*) is called the *inner product* between the vectors **x** = (*x*_1_, *x*_2_, *…* , *x*_*n*_) and **y** = (*y*_1_, *y*_2_, *…* , *y*_*n*_), that is defined as

$$\varphi \left(x,y\right)=\left(x_{1},x_{2},\ldots ,x_{n}\right)\cdot \left(y_{1},y_{2},\ldots ,y_{n}\right)=x_{1}y_{1}+x_{2}y_{2}+\cdots +x_{n}y_{n}.$$ (1)

Usually the inner product of two vectors **x**, **y** is dented with the angular bracket ⟨**x**, **y**⟩. In the Euclidean space $R^{n}$ the distance between the points whose coordinates are given by the vectors **x** and **y** is

$$d\left(x,y\right)=\sqrt{\left\langle x,y\right\rangle }=\sqrt{\varphi \left(y-x,y-x\right)}=\sqrt{\left(y_{1}-x_{1}\right)\left(y_{2}-x_{2}\right)\cdots \left(y_{n}-x_{n}\right)}\equiv =\|y-x\|.$$ (2)

To embed a graph into an Euclidean space, we used the classical (metric) multidimensional scaling algorithm that, given as an input the pairwise dissimilarities matrix {*d*_*ij*_}, reconstructs a map that preserves distances. The algorithm implements the following steps.

1. Find a random arrangement of points, for example, by taking a sample from a normal distribution.
2. Determine the distances between the points.
3. Find the best monotonic transformation for the proximity to get the best scaled data.
4. Find a new arrangement of points to reduce the stress between the optimally scaled data and the distances. The stress of the embedding in Euclidean space is defined by the following residual sum of squares

$$Stress\left(x_{1},x_{2},\ldots ,x_{n}\right)=\sqrt{\underset{i\neq j=1,\ldots ,n}{\sum }{\left(d_{ij}^{\left(\text{input}\right)}-d_{ij}^{\left(\text{embedding}\right)}\right)}^{2}}$$ (3)

where, in the case of Euclidean embedding, $d_{ij}^{\left(\text{embedding}\right)}=\|x_{i}-x_{j}\|$ .

5. Compare the stress to a certain standard. If the stress is too low, stop the algorithm; otherwise, go back to step 2.

We implemented the embedding in R (R Core Team, 2021), using the function cmdscale (Gower, 2015) of the library stats. Theoretical foundations and details about multidimensional scaling techniques can be found in many text books and review paper [see, for example, (Borg and Groenen, 2005; Cox and Cox, 2008; Zhang and Takane, 2010)].

### 2.2 Hyperbolic geometry and the Poincaré model

Hyperbolic geometry accepts the first four axioms of Euclidean geometry but rejects the fifth, namely, that there exists a line and a point not on the line with at least two parallels to the given line crossing through the provided point. This is equivalent to performing geometry on a surface with a constant negative curvature. This geometry differs greatly from the more conventional Euclidean geometry, and are hard to visualise. The main reason is that by the Hilbert’s theorem (Hilbert, 1933) the hyperbolic plane cannot be isometrically embedded into Euclidean 3D-space (isometric means preserving the length of every curve). We must flatten the curvature to display the hyperbolic plane. In doing this, many of the straight lines in hyperbolic space end up being curved as a result. The French mathematician Henri Poincaré is responsible for one of the widely accepted theories for flattening the hyperbolic plane and the *n*-dimensional ball model (Poincaré disk in 2D) (Anderson, 2005).

**FIGURE 2:**
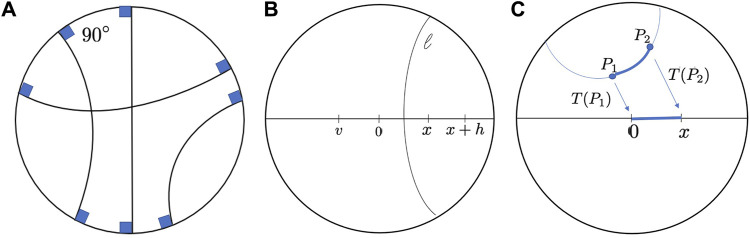
**(A)**. Geodesics in Poincaré disk. **(B)**. Reflections in Poincaré disk geometry. 0, *x*, *x* + *h* and *v* are points on the diameter. 0 is the reflection of *x*, and *v* is the reflection of *x* + *h* with respect to the hyperbolic segment *l*. **(C)**. The distance between two generic points *P*_1_ and *P*_2_ can be found first transforming *P*_1_ tp 0 and *P*_2_ to *x*.

The Poincaré *n*-dimensional ball $B_{R}^{n}$ $\left(B_{R}^{n}=\left\{x\;|\;\|x{\|}^{2}<1\right\}\right)$ is a model for *n*-dimensional hyperbolic geometry in which lines are represented by circle diameters or by arcs of a circle with ends perpendicular to the boundary of the ball. (Figure 2). If *n* = 2 the Poincaré model is a unit open disc. We briefly summarize here the method in Conn (2010) to calculate the distances in the unit disc model Consider the fractional linear transformation *S* that sends *∞*↦*i* and ±1↦ ± 1. *S* sends the real axis to the boundary of the unit disc and, since fractional linear transformations preserve the orientation of circles, it sends the upper half-plane to the disc’s interior. The *H*_2_-distance between two points *a*, *b* in the unit disc is the *H*_1_-distance between their preimages *S* ^−1^(*a*), *S* ^−1^(*b*) in the upper half-plane (Conn, 2010), and in this way the unit disc inherits a metric from the metric of the upper half-plane.

Let *D* ^1^ denote the interior of the unit disc and suppose

$$\gamma :\left[0,1\right]\to D^{1}$$

is a piecewise continuously differentiable curve. If *H*_*k*_(*γ*) (*k* = 1, 2) denotes the length of the curve *γ*, then

$$H_{2}\left(\gamma \right)=H_{1}\left(S^{-1}◦\gamma \right).$$ (4)

Writing *S* ^−1^ ≡ *T*, we have

$$\begin{array}{cc} H_{1}\left(T◦\gamma \right) & =\int_{T◦\gamma }\frac{1}{Im\left(z\right)}|dz|=\int_{0}^{1}\frac{1}{Im\left(\left(T◦\gamma \right)\left(t\right)\right)}\left|{\left(T◦\gamma \right)}^{\prime }\left(t\right)\right|dt\\ & =\int_{0}^{1}\frac{1}{Im\left(T\left(\gamma \left(t\right)\right)\right)}\left|T^{\prime }\left(\gamma \left(t\right)\right)\right|\left|\gamma^{\prime }\left(t\right)\right|dt=\int_{\gamma }\frac{1}{Im\left(T\left(z\right)\right)}\left|T^{\prime }\left(z\right)\right||dz|. \end{array}$$ (5)

Since *T* has the form

$$T\left(z\right)=\frac{iz-1}{-z+i}.$$ (6)

we have that

$$Im\left(T\left(z\right)\right)=Im\left(\frac{\left(iz-1\right)\left(-\bar{z}-i\right)}{|-z+i{|}^{2}}\right)=\frac{1-|z{|}^{2}}{|-z+i{|}^{2}},$$ (7)

and

$$\left|T^{\prime }\left(z\right)\right|=\frac{2}{|-z+i{|}^{2}},$$ (8)

we obtain

$$H_{2}\left(\gamma \right)=\int_{\gamma }\frac{2}{1-|z{|}^{2}}|dz|$$ (9)

that is general formula calculating distances in the Poincaré disc. $2/\left(1-|z{|}^{2}\right)dz$ is the element of arc length. Consequently, the distance between two points a,b∈C on Poincaré disc is

$$d\left(a,b\right)=H_{2}\left(a,b\right)=\log\frac{\left|1-\bar{a}b|+|b-a\right|}{\left|1-\bar{a}b|-|b-a\right|}.$$ (10)

Any diameter of the unit disc is a geodesic, so if *z* is a point in the unit disc, then the Euclidean segment from 0 to *z* is also a hyperbolic segment from 0 to *z*. We have hence that

$$H_{2}\left(0,z\right)=\int_{0}^{\left|z\right|}\frac{2}{1-t^{2}}dt=2\tanh^{-1}\left(\left|z\right|\right)=\log\left(\frac{1+|z|}{1-|z|}\right).$$ (11)

Complex networks connect different nodes. This diversity indicates that there is at least some element taxonomy, meaning that all nodes can be classified in some way. This classification means that nodes can be separated into large groups that are made up of smaller subgroups that are made up of even smaller sub-subgroups, and so on. The relationships between such groups and subgroups can be approximated by treelike structures, which illustrate hidden hierarchies in networks. Krioukov et al. demonstrated that the metric structures of trees and hyperbolic spaces are equivalent (Krioukov et al., 2010; Kurkofka et al., 2021; Lecca, 2023; Lecca and Re, 2022). It is not necessary for the node classification hierarchy to be exactly a tree, but rather approximately a tree. When a network can be approximated by a tree, its latent geometry is negatively curved (Gromov, 2007).

To perform the embedding into a hyperbolic space (Poincaré model), we used the function hydraPlus of the R library hydra (HYperbolic Distance Recovery and Approximation) (Keller-Ressel, 2019), that uses a strain-minimizing hyperbolic embedding based on reduced matrix eigendecomposition (Keller-Ressel and Nargang, 2020). The stress of embedding in hyperbolic space is then given by formula (3), where *d* ^(embedding)^ is given by the output of hydra.

### 2.3 Spherical geometry and embedding

Spherical geometry is the geometry of a hypersphere’s surface. The hypersphere can be easily immersed in euclidean space; for example, the embedding of a three-dimensional sphere of radius *r* is well known relation *x* ^2^ + *y* ^2^ + *z* ^2^ = *r* ^2^, with **x** = (*r* sin *u* sin *v*,*r* cos *u* sin *v*,*r* cos *v*)^*T*^. A simple extension of this is the embedding of a (*n* − 1)-dimensional sphere in *n*-dimensional space:

$$\overset{n}{\underset{i=1}{\sum }}x_{i}^{2}=r^{2}.$$ (12)

There is a constant sectional curvature of 1/*r* ^2^ throughout this curved surface. The length of the shortest curve that lies in the space and connects the two points is the geodesic distance between two points in a curved space. The geodesic on the hypersphere is a perfect circle for a spherical space. The distance is equal to the width of the arc that connects the two locations on the great circle.

If two points in the hypersphere’s centre form an angle with *θ*_*ij*_, then the distance between them is

$$d_{ij}=r\theta_{ij}.$$ (13)

A point can be represented by a position vector **x**_*i*_ of length *r* with the coordinate origin at the origin of the hypersphere. We can also write

$$d_{ij}=r\;\cosh\frac{\left\langle x_{i},x_{j}\right\rangle }{r^{2}}$$ (14)

since the inner product is ⟨**x**_*i*_, **x**_*j*_⟩ = *r* ^2^ cos *θ*_*ij*_.

To perform the embedding of the graph in a hypersphere, we used the method proposed by Wilson et al. (2014a), and the Matlab code that this authors made available in Wilson et al. (2014b). We summary briefly the core of embedding method in this way.

Given a dissimilarity matrix *D*, we want to determine the set of points on a hypersphere that give the same distance matrix. Because the curvature of the space is unknown, we must also determine the radius of the hypersphere. We have *n* items of interest, thus we would ordinarily look for a *n* − 1 dimensional Euclidean space. A coordinate system with the origin at the centre of the hypersphere is considered. A matrix **X** of point positions vectors is constructed in such a way that

$$XX^{T}=Z=\left\{z_{ij}\right\}=r^{2}\cos\frac{d_{ij}}{r}$$ (15)

**Z** is a *n* × *n* matrix that is positive semi-definite and has rank *n* − 1 since the embedding space has dimension *n* − 1, **X** is made up of *n* points that are located in a space of dimension *n* − 1, and so does the embedding space. This means that the **Z**’s eigenvalues are positive, with only one being zero. This observation can be used to calculate the radius of curvature. Then, in order to find *r*, Wilson et al. (2014a) proposed to create **Z**(*r*) and identify the smallest eigenvalue *λ*_1_, to calculate then the optima radius of curvature as

$$r^{*}=\arg\underset{r}{\min}|\lambda_{1}\left[Z\left(r\right)\right]|.$$ (16)

The stress of embedding in hyperbolic space is then given by formula (3), where *d* ^(embedding)^ is given by the elements of the matrix **Z**.

### 2.4 How latent geometry influences network dynamics

By the term “dynamic” of a system, we mean the *time and space* evolution of the system as described by differential and/or algebraic equations whose variables are quantitative features of the system’s actors, and whose mathematical form model the topological system’s organization. The equations of the dynamics are parameterized by the dynamical properties of the system itself (such as frequency of oscillation, if the system is oscillatory, elastic constant, if the system is assimilated to a spring system, specific rate of reaction, *etc.*) There are interesting studies showing how the geometry of complex networks affects the dynamics. To cite a relevant contribution to the field, we mention the work of Millán et al. (2018) which shows that the latent geometry of a network has a significant impact on the synchronization dynamics. Unlike Millán et al. work, which is more focused on the dynamic properties of the system (i.e., parameters and synchronization laws), here we focus on the influence that latent geometry can have on network organization. And since from the network organization, the dynamics of the network is derived, we can expect latent geometry to influence the dynamics. In particular, the geometry of the network determines the presence or absence of functional modules containing highly cooperative nodes. The identification of these possible functional clusters can be done correctly only if the metric space of the network is identified. In fact, this space defines the distance between nodes, the measure on which clustering algorithms are based. A clustering in Euclidean space may lead to a different result from clustering in hyperbolic space, the distance computed in this space being different from the distance computed in Euclidean space. The correct dynamics is one whose parameters and functional modules are established by the latent geometry for at the network under consideration.

In this study, we conceived a network as a spring system. Through the identification of the most appropriate latent geometry of the network under consideration, i.e., that geometry that most closely reproduces the values of the spring constants of the edges thought of as springs, we were able to identify cluster of gene drivers for the network dynamics. The role of drivers of these genes was validated through functional analysis of them. In the next sections, the data from which we built the network, as well as the model and analysis of the network itself are reported.

## 3 Data and gene network

We use here the data of gene expression relevant to the landscape of Chronic Myeloid Leukemia K562 cells. We refer the reader to a recent publication by the authors (Lombardi et al., 2022)], where we describe the experimental activity implemented for data measurement and algorithmic procedures for selecting differentially expressed genes. For the reader’s convenience we summarise it briefly below.

On an Agilent whole human genome oligo microarray (#G4851A, Agilent Technologies, Palo Alto, CA), the RNAs from the samples were hybridised. This microarray consists of 60,000 distinct human transcripts represented by 60-mer DNA probes created using SurePrint technology. The manufacturer’s recommended protocol was followed when one-color gene expression was carried out. In a nutshell, samples were used to extract the total RNA fraction using the Trizol Reagent (Invitrogen). Agilent Technologies’ Agilent 2100 Bioanalyzer was used to evaluate the quality of the RNA samples. RNAs with low integrity (RNA integrity number less than 7) were not included in the microarray analysis. Using the Low Imput Quick-Amp Labelling Kit, one colour (Agilent Technologies) in the presence of cyanine 3-CTP, labelled cRNA was produced from 100 ng of total RNA. In a revolving oven, hybridizations were carried out for 17 h at 65°C. Agilent’s scanner produced images with a 3 *μ*m resolution, and Agilent Technologies’ Feature Extraction 10.7.3.1 software was utilised to extract the microarray raw data. The GeneSpring GX 11 programme (Agilent Technologies) was then used to analyse the microarray results. Data transformation was used to normalise all of the data’s negative raw values to 1.0 using the 75th percentile. Only the probes expressed in at least one sample (marked as Marginal or Present) were retained using a filter on low gene expression.

The data used in this work come from the aforementioned examination of the CML cell transcriptome (K562) using microarray hybridization under various settings. The cells were transfected with full-length PTPRG and compared to three controls: cells transfected with the empty vector, cells transfected with a PTPRG inactive mutant with a mutation in the catalytic domain (D1028A), and cells treated with Imatinib, which targets the oncogene BCR/ABL1. The complete dataset is publicly available at the GitLab repository. https://gitlab.inf.unibz.it/Paola.Lecca/chronic-myeloid-leukemia-genes.

Here, from the entire dataset available at this link, we only considered the gene expression levels of the untreated group (empty vector and inactive mutant domain D1028A) and those of the treatment group expressing full-length PTPRG. We then selected the genes, that, according to the analysis in Lombardi et al. (2022), result to be differentially expressed between the two groups. To construct the gene network, we queried PathwaysCommons (PathwayCommons.All.hgnc repository) (Cerami et al., 2010a; Cerami et al., 2010b) by providing as input for the search the list of gene names we considered in this study. The obtained gene network is a representation of molecular associations specified through nodes (genes) and edges (molecular interactions or statistical relationships). Among the various format, PathwaysCommons gives as output result of the query the gene networks also in SIF (Simple Interaction format), which is a table providing details on gene-gene interactions. This format offers various levels of detail such as: interaction type, reference data source, Pubmed id, reference pathways, and mediators id. Our analyses focused on the most granular level of information, namely, the interactions between pairs of genes, listed in the SIF table as“Participant A” and “Participant B” (we refer the reader to the public repository of our data to view the data format). The types of interactions included in the network are as follows: interacts-with, in-complex-with, catalysis-precedes, controls-state-change-of, controls-transport-of, controls-transport-of-chemical, controls-expression-of, controls-phosphorylation-of, controls-production-of, chemical-affects, consumption-controlled-by.

As a final result of querying to common pathways and selecting differentially expressed genes on the two groups (treated and untreated), we obtained a network that is a non-planar multi-edge graph with 2,080 nodes and 3,745 edges, that we simplify to a non-multi-edge graph with 2,080 nodes and 3,464 edges.

### 3.1 Graph embedding in presence of noise of input data: Some remarks

The presence of noise on the data in the adjacency matrix used as input to the graph embedding procedures could be a vexing problem if embedding stresses in different metric spaces are to be compared to identify which metric space is best representative of the latent geometry of the network. Noise, for example, may not allow weak edges to be distinguished from the absence of nodes and may affect the reliability of the measurement of even the most robust arcs (i.e., those with the greatest weight). Data analysis frequently faces the challenge of distinguishing between real weak edges and noise-induced low-weight edges. To solve this issue, noise is typically either eliminated or studied in the absence of data.

In the specific case of our study, the experimental data from which we start to construct the weighted adjacency matrix of the graph are very accurate. Our dataset was validated comparing the outcome of the cDNA microarray with the analysis of a specific set of genes chosen for being informative and for being predicted up and downregulated. Validation was performed in triplicate with quantitative PCR on a new, independent, preparation of cDNA derived from the same cell lines, thus ensuring that the results present in our dataset represent a true variation in mRNA levels. Notably the analysis permitted to predict a shift to erythroid differentiation of the cells that was confirmed also at protein level. All supporting data are reported on the publication (Lombardi et al., 2022).

Interesting and noteworthy works elucidating the role and the influence of noise in graph embedding has been done recently by Maddalena et al. (2022) and Okuno and Shimodaira (2019). The treatment of the presence of noise is in fact so complex that it deserves the implementation of a focused study and consequently the writing of a separate article. It is out of the scope of this study, give the high quality of the data we used here, but it is our intention to explore this issue further in a forthcoming study.

To the best of our knowledge at present, we find of particular interest the study of Blevins et al. (2021). Instead, by analysing the structure of noisy, weak edges that have been artificially added to model networks, the authors explored how noise and data coexist in this work. They discovered that there are qualitative classifications of noise structure that arise, and that these noisy edges can be used to categorise the model networks. The authors state that the structure of low-weight, noisy edges varies depending on the topology of the model network to which they are added. Interestingly, Blevins et al. showed that noise is a complex, topology-dependent, and even valuable phenomenon in characterising higher-order network interactions rather than a monolithic annoyance.

## 4 Mathematical model of the gene network

**FIGURE 3:**
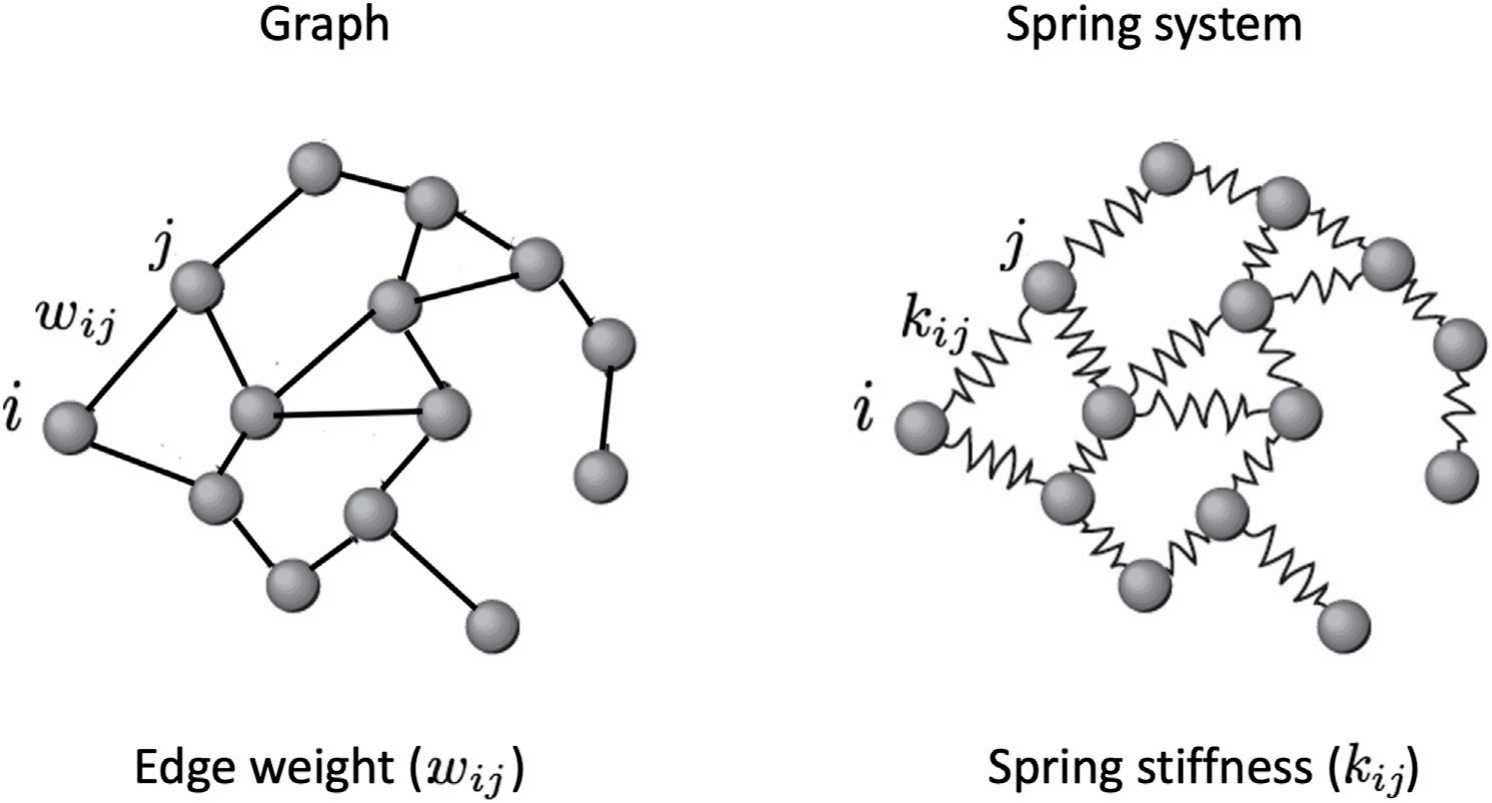
A spring system, also known as a spring network, is a model of physics used in engineering and physics that is represented as a graph with a mass at each vertex and a spring with a specific stiffness and length along each edge. Extending the Hooke’s law to higher dimensions (see the Section ?), it is possible to calculate the spring stiffnesses that in this model represent the edge weights.

To estimate the weights of the network arcs, we conceptualise the network as a system of masses (representing the nodes) and springs (representing the edges), as in Figure 3. Estrada and Hatano (2010) has brought a remarkable contribution to spring-like network models. In a complex network, Estrada and Hatano suggested a new metric for measuring node vulnerability. The metric is based on an analogy where the network’s nodes are represented by masses and its edges by springs. They defined the measure as the node displacement, or the amplitude of vibration of each node, under variation caused by the thermal bath in which the network is intended to be immersed, and that represents the environment from which stimuli may possibly come. The Estrada index for the vibrational centrality of the node *i* is defined as the node displacement (Δ*x*)_*ii*_

$${\left(\Delta x\right)}_{ii}=\sqrt{\frac{T}{k}L_{ii}^{+}},$$ (17)

where *T* is the temperature of the external bath, and *k* is the spring stiffness. Estrada and Hatano assumed that the network edges are identified with springs with a common spring stiffness *k*.

Instead in the network spring model of Lecca and Re (2020), the authors postulated that weights of the arcs are given by the stiffness of the springs representing the arcs, so each arc may have a different stiffness/weight. The harder the spring, the more efficiently the signal is transmitted from node to node; the softer the spring, the less quickly the signal is transmitted from node to node. According to this metaphor, edges characterised by high values of the stiffness of the hypothetical spring joining them are nodes that interact more effectively than nodes whose spring stiffness joining them is lower. The stiffness of the spring is thus interpreted as the efficiency of the interaction. Next, we briefly summarize the computational method developed by Lecca and Re (2020), and used in this study, to calculate the stiffnesses of the springs.

The elastic force applied to the nodes by the springs according to the generalised Hooke’s law for a system of *N* springs is

$$F_{\text{elastic}}=-K\Delta x,$$ (18)

where ***K*** is the matrix providing the stiffnesses of all of the springs, and the elements of Δ ***x*** are the vibrational centralities of the nodes. We obtain the force on the nodes by multiplying *F*_elastic_ by the transpose of the graph weighted incidence matrix ***C*** ^*⊤*^, where, in general, the weights are given by the node masses, i.e.,

C=AM (19)

where ***A*** is the unweighted incidence matrix. We should remark that weighting the incidence matrix with node mass values means taking into consideration the nodes’ inertia to the propagation of the elastic force through the springs incident to them (Lecca and Re, 2020). In this study, the nodes’ masses are given by the nodes’ total degree.

The force on node is then defined by

$$F_{\text{nodes}}=-C^{⊤}K\Delta x$$ (20)

At the equilibrium *F*_nodes_ = **0**, i.e.,

$$C^{⊤}K\Delta x=0,$$ (21)

where ***K*** is obtained as the nullspace (or kernel) of ***C*** ^*⊤*^, in formula:

$$K=\text{Ker}\left(C^{⊤}\right).$$ (22)

Indeed, all vectors ***K*** that have the properties that ***C*** ^*⊤*^ ***K*** = 0 and ***K*** are not zero make up the null space of any matrix ***C*** ^*⊤*^.

Once **K** is obtained, we construct the dissimilarity matrix of the graph, which is then used as input for the embedding algorithms, as follows

$$d_{ij}=\frac{1}{1+k_{ij}}$$ (23)

where *k*_*i*_ *j* are the elements of the matrix **K**. Thus, nodes connected by a spring with a high value of the elastic constant have a lower dissimilarity value than nodes connected by a spring with a low value of the elastic constant. This reflects the situation where the propagation speed of the interaction along a spring with high stiffness is higher than along a spring with low stiffness.

Of particular interest is in case the system is not in equilibrium. In fact, ***K*** is independent on Δ*x* only when the system is at equilibrium, i.e., when *F*_nodes_ = **0** and Eq. 21. In non-equilibrium conditions, we have instead that *F*_nodes_ = ***C*** ^*⊤*^ ***K*** Δ ***x*** ≠ **0**. Suppose that we know the forces *F*_nodes_ acting on nodes. For example, this could be the case in which perturbation experiments are implemented to measure and analyse the responsiveness of the network nodes to stimuli and/or stresses, or, assimilating forces on nodes to white noise distributed over all nodes, noise always present in biological systems at the micro-scale given their inherent stochastic dynamics). To calculate the matrix ***K***, in this case, the requirements are that the matrices ***C*** ^*⊤*^ and Δ*x* are invertible, so that

$$K={\left(C^{⊤}\right)}^{-1}F_{\text{nodes}}{\left(\Delta x\right)}^{-1}.$$ (24)

Note, Δ*x* is invertible if and only if all the entries on its main diagonal are non-zero, which means that little to much all nodes have a significantly non-zero response to stress.

## 5 Results

**FIGURE 4:**
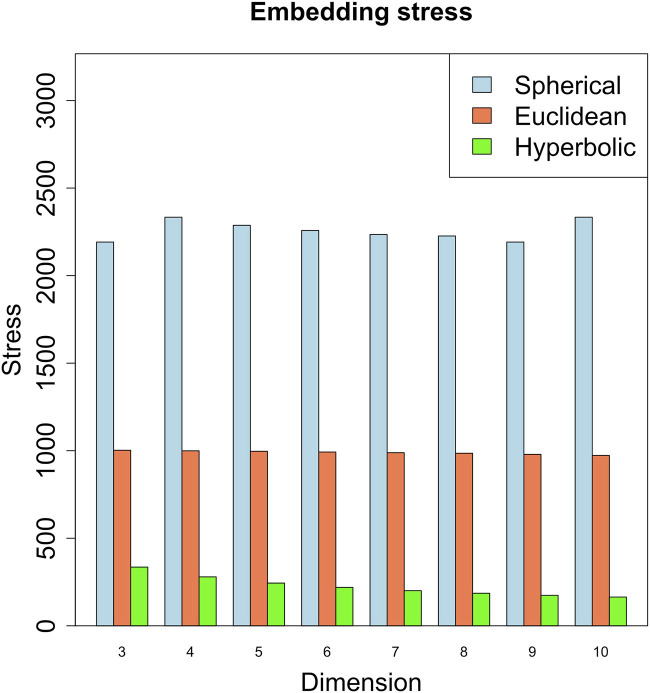
Embedding stress vs. metric space dimensions. The embedding with the least stress is the hyperbolic one, revealing a putative hyperbolic latent geometry of the gene network.

We embedded the gene network in the three metric spaces considered by considering different dimension values. We started with dimension 3, since the network is not planar. As shown in Figure 4, the embedding that produces the least amount of stress on the dissimilarity matrix - obtained as in Section 4 - is the hyperbolic embedding. The network is then characterized by a power-law degree, and by a hierarchical structure reflected also in the presence of clusters in the radial coordinates of the points, that is known to represent the node popularity (Papadopoulos et al., 2012; Yang and Rideout, 2020; Kovács and Palla, 2021). By nodes having high popularity, we mean nodes that are related to the majority of the other nodes in the graph [see also (Lecca et al., 2023) for a short review of the Papadopoulos et al. definition of node popularity]. These nodes can aid in the efficient spreading of information throughout the network.

**FIGURE 5:**
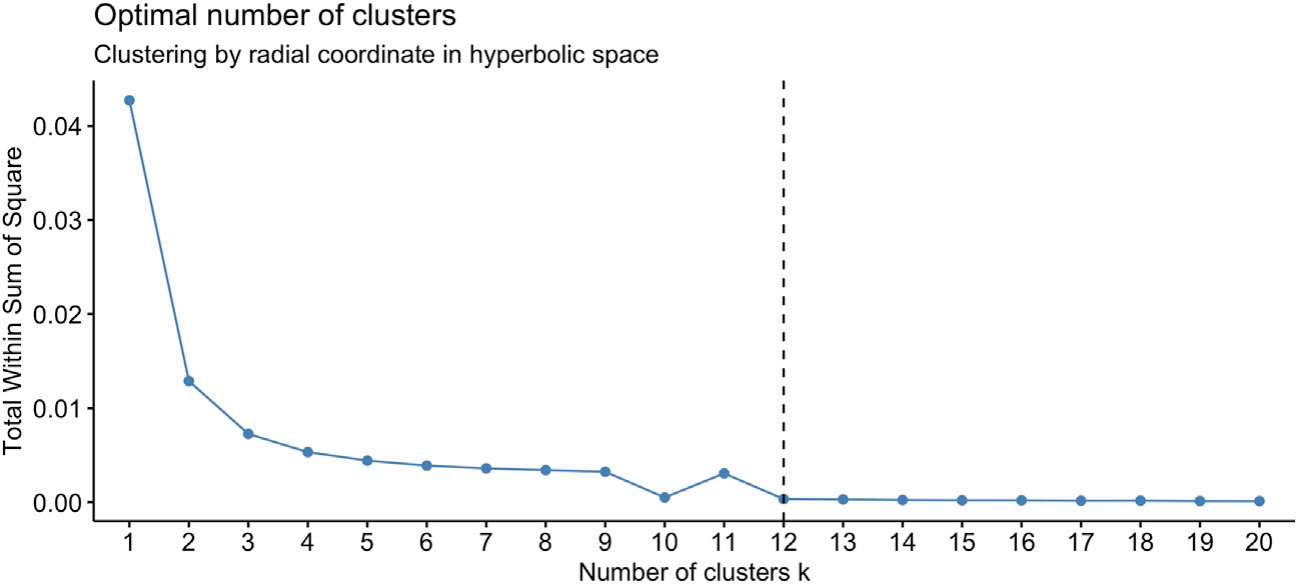
The optimal number of clusters of the set of radial coordinates of the points (node) on the Poincaré disk, according to the Elbow method, is 12.

**FIGURE 6:**
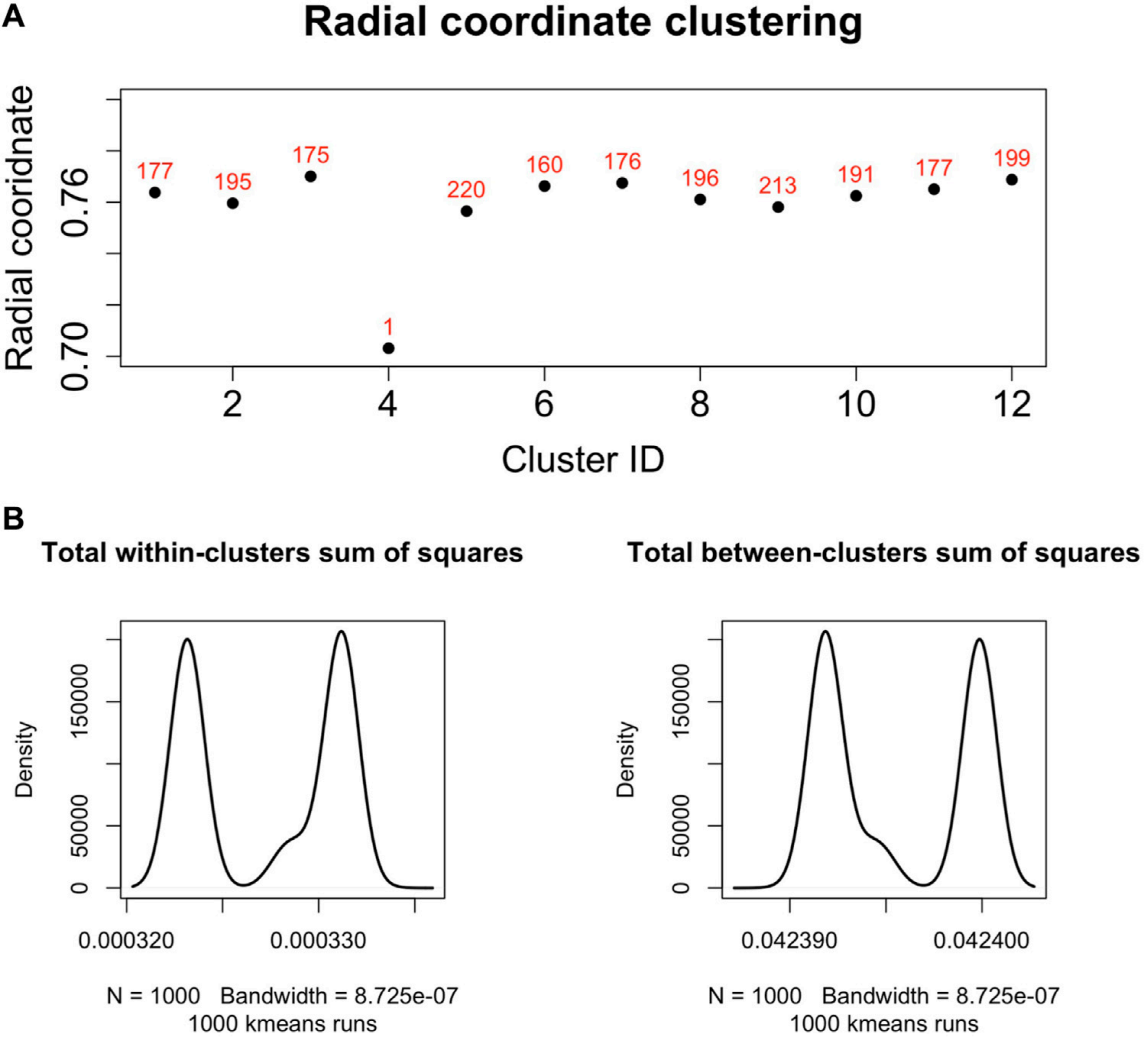
**(A)**. Centroids of the radial coordinate clusters *versus* cluster identifier in a single run of k-means algorithm. The number of genes belonging to each cluster is shown in red. The gene belonging to the cluster number four is ZRANB1, a gene characterized by low immune cell specificity, and belong to NK-cells immune cell expression cluster (Uhlén et al., 2022b). **(B)**. We performed 1,000 runs of the k-means for the clustering of the radial co-ordinate of the nodes of the network in hyperbolic space and drew the distributions of the within- and between-clusters sum of squares. The within-cluster sum of squares quantifies the internal cohesion inside each cluster. The between-cluster sum of squares quantifies the external separation between clusters. The figure shows that for the k-means clustering of the radial coordinates the between-clusters sum of squares is two orders of magnitude greater than the within-cluster sum of squares, revealing the accuracy and then reliability of the clustering results.

We found that the set of radial coordinates, whose values range in [0.7036133, 0.7709305], is characterized by 12 clusters as determined by the Elbow method (Umargono et al., 2020) (see Figure 5). The range of radial coordinate values is. In Figure 6A we report the cluster ID and the size of the 12 clusters of the radial coordinates as obtained by a single run of the k-means algorithm. We found that the gene with the smallest popularity (i.e., with the smallest radial coordinate) is ZRANB1. This gene allows for K63-linked polyubiquitin modification-dependent protein binding and thiol-dependent deubiquitinase activity. Involved in a variety of functions, including the positive control of the Wnt signalling pathway, protein deubiquitination, and cell morphogenesis regulation (NIH, 2023). However, according to the date in The Human Atlas of Proteins is a low immune cell specificity gene (Pontén et al., 2008; Uhlen et al., 2017; Uhlén et al., 2022a; Uhlén et al., 2022b). To assess the stability and the quality of the clustering, we repeated the k-means 1,000 times and graphed the distributions of the within- and between-sum of squares (see Figure 6B) that show that the first is two order of magnitude smaller then the second. The skewness of the distributions and the disproportion between within- and between-cluster sum of squares indicate the stability and accuracy of the clustering, respectively.

Nodes with similar radial co-ordinate have similar popularity, so clustering according to the radial co-ordinate identifies communities of nodes with similar popularity. However, the radial co-ordinate, in addition to representing the popularity of a node, i.e., its degree of connectivity with other nodes in the network, identifies the distance from the origin in the Poincaré ball. In a network with hyperbolic latent geometry, in its representation in the Poincaré ball, the mean degree of a node is a negative exponential function of the node’s radial coordinate (Krioukov et al., 2010). Thus, the average degree of a node decreases exponentially with increasing distance of the node from the origin of the Poicaré ball, or, in other terms, the higher the radial co-ordinate of a node, the lower its degree on average. The area inside the unit ball represents the infinite hyperbolic plane, and, consequently, nodes with radial co-ordinate equal 1 are points at infinity. Clustering according to the radial co-ordinate thus identifies bands of points (nodes) that are concentric on the Poincaré disc and that have a decreasing degree of connectivity as one moves away from the origin. This is why we say that clustering according to radial co-ordinates allows clusters of strongly interconnected nodes to be identified (if any). The cluster of nodes closest to the origin identifies not only nodes with high connectivity, but also nodes that are close to each other (this second characteristic also applies to clusters far from the origin). The coexistence of two characteristics such as high degree and small distance between nodes is typical of a cluster of nodes with efficient interactivity and greater inertia to perturbations induced by external stimuli, such as variation of expression level, interactions with drugs, *etc.* The short inter-node distance reflects the high efficiency of communications, the high connectivity may be responsible of the node robustness.

Nodes that are thus highly interconnected and close in network metric space are potential drivers of network dynamics. This conjecture is demonstrated in the case where the distribution of the stiffness of the arcs in the cluster to which these nodes belong is similar to the distribution of the stiffness of the arcs in the overall network. Stiffness is in fact a dynamic property of the system. The cluster of nodes and arcs with dynamic properties that are reflected in the dynamic properties of the entire network can thus be considered a cluster of driver nodes, a characteristic that designates it as a prime candidate for further wet experiments. In the case of our study, experiments and data from the literature support the hypotheses formulated by the computational analysis, as we shall see below. Indeed, the results we report below are intended to demonstrate these statements.

**FIGURE 7:**
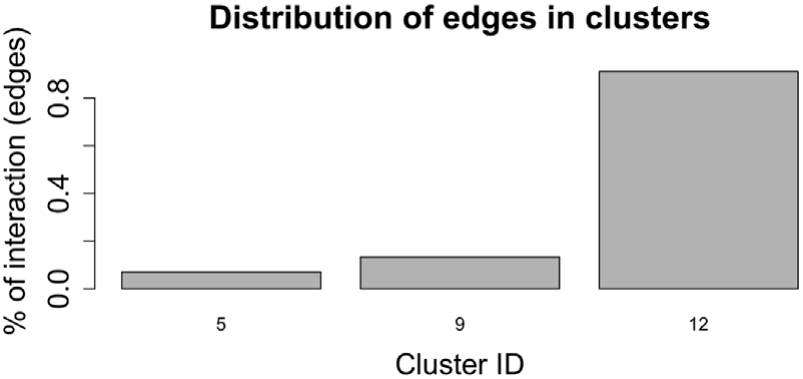
Barplot showing the percentage of edges joining nodes belonging to the same cluster according to the radial co-ordinate value. Only clusters 5, 9 and 12 contain nodes that are connected by an edge and belong to the cluster. The remaining edges connect nodes belonging to different clusters.

**FIGURE 8:**
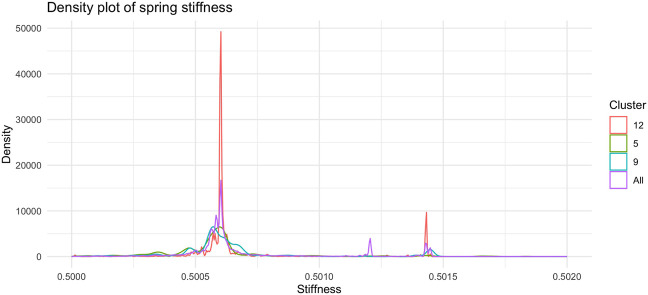
Density plot of the distribution of spring stiffness in the tree clusters (5, 9, and 12) and in whole dataset. The trend of the red curve for Cluster 12 is the one that most accurately reproduces the global density plot of the stiffness of the arches of the entire network. The nodes in Cluster 12 recapitulate the overall dynamics of the network through their interactions and can therefore be regarded as putative drivers of the dynamics.

Figure 7, shows the barplot of the percentage of edges connecting nodes belonging to the same cluster of radial coordinates. Of the 2,162,160 total edges of the graph 1,512 belong to cluster 5, 2,877 to cluster 9, and 19,701 to cluster 12. The remaining 2,138,069 arcs connect nodes belonging to different clusters. In order to understand whether and, if so, how clustering according to radial co-ordinate is reflected in the distribution of spring stiffness, we produced the graph in Figure 8, showing the density plots of the spring stiffness of the interactions between node within the three clusters (5, 9 and 12) compared with the density plot of all spring stiffness of the network. To make the results easier to read and understand, we rescaled the spring stiffness values obtained by formula (22) within a range between 0 and 1 and applied formula (23) to the values obtained in this range.

Of interest we find as shown in this Figure 8 the two peaks of the density plot in red colour corresponding to the stiffness of the interactions between the nodes belonging to cluster number 12. Of the three clusters of radial node distance, number 12 is the one that best reflects the density plot of total spring stiffness. The interactions between nodes belonging to cluster 12 are markedly clustered as is the distribution of stiffnesses across all the arcs of the graph. We interpret this result as the fact that cluster 12 contains nodes that share similar popularity values and are involved in driver interactions of the network dynamics, since the distribution of spring stiffnesses of the arcs of these nodes reproduce the distribution of spring stiffnesses of the entire network.

**FIGURE 9:**
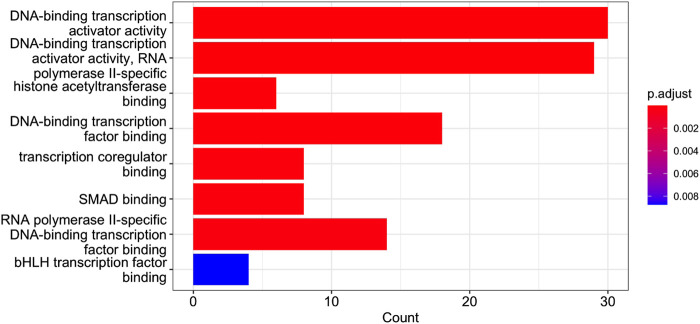
GO Enrichment Analysis of the gene set of Cluster 12. The barplot shows the enrichment GO categories of molecular functions after false discovery rate control. See also the verbose tabular outbut in Cluster_12_GSEA_results_EnrichGO_MF.xlsx provided in Supplementary Material.

**FIGURE 10:**
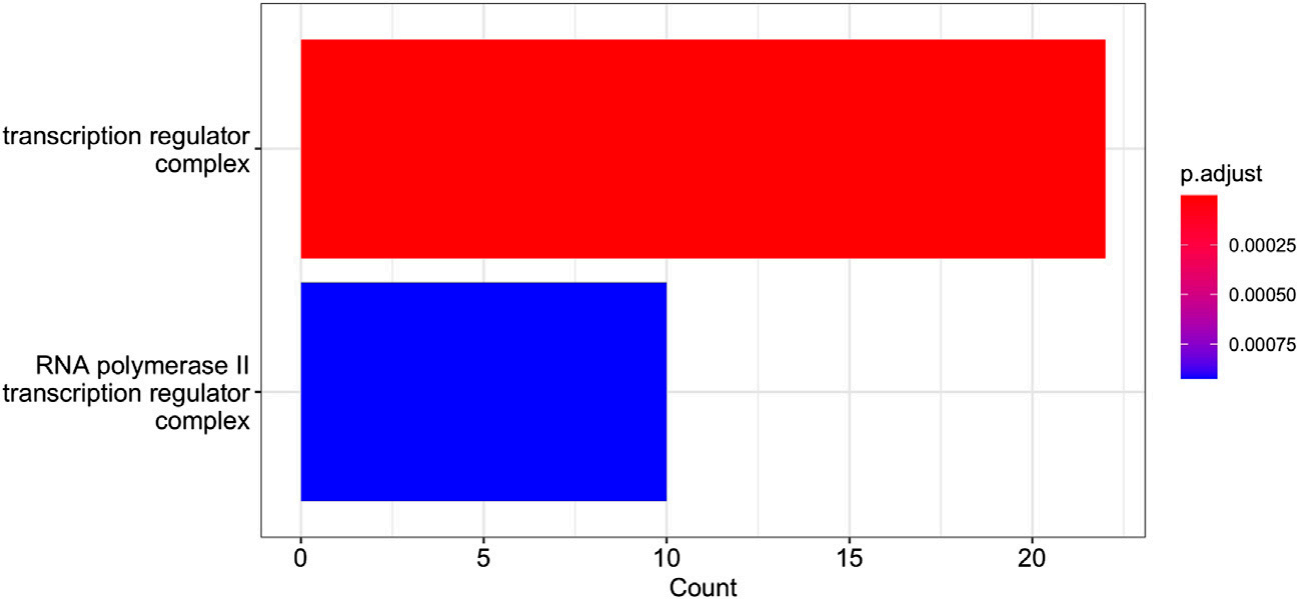
GO Enrichment Analysis of the gene set of Cluster 12 (obtained with the R function enrichGO). The barplot shows the enrichment GO categories of cellular compartment after false discovery rate control. See also the verbose tabular outbut in Cluster_12_GSEA_results_EnrichGO_CC.xlsx provided in Supplementary Material.

**FIGURE 11:**
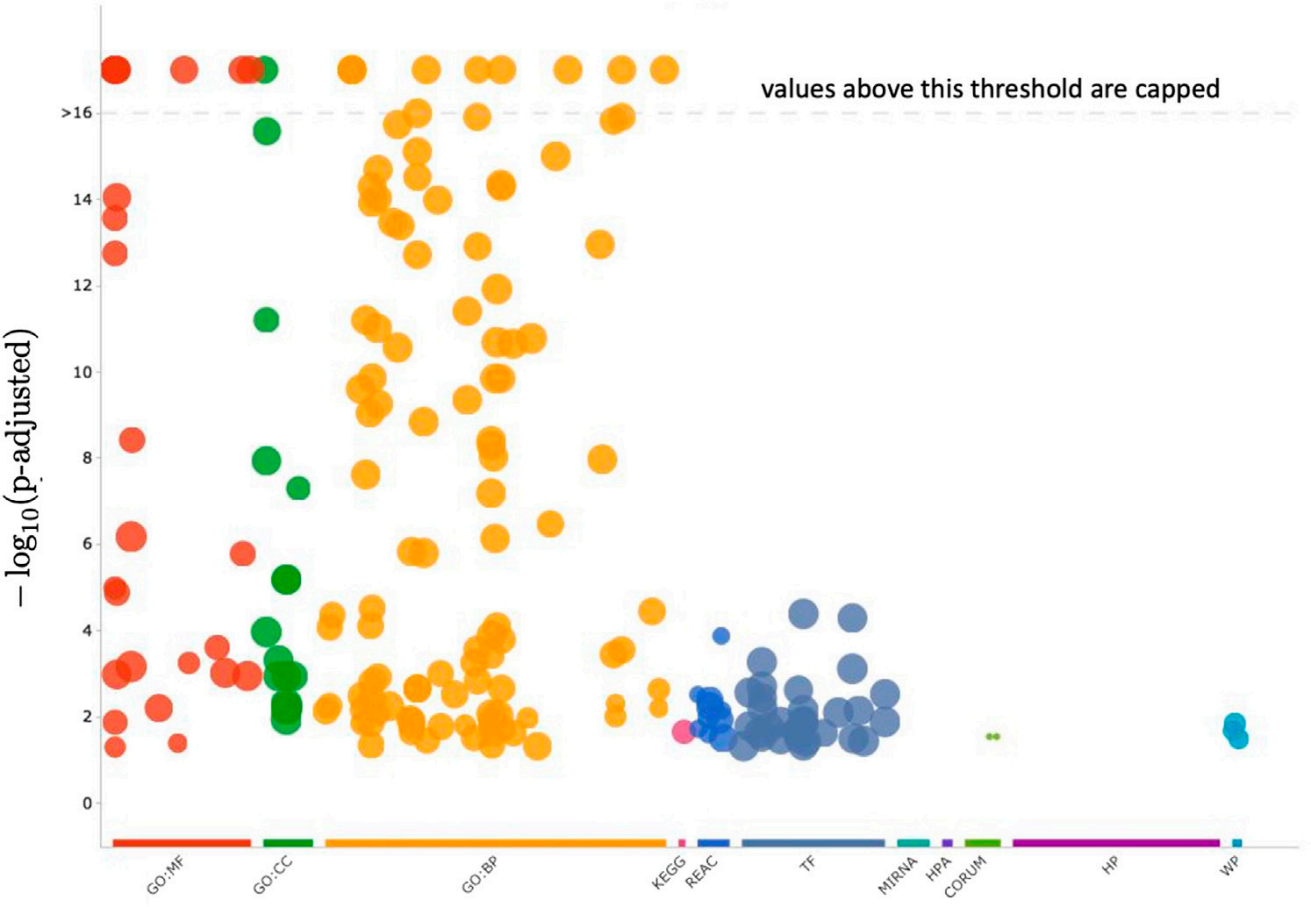
In this figure, the enrichment results of the gene set of Cluster 12 are visualized with a Manhattan-like-plot using the function gostplot (Raudvere et al., 2023). The *x*-axis depicts functional terms that are colour-coded and categorised according to data sources and positioned in the fixed “source_order.” The order is set up so that terms that are close together in the source hierarchy are also close together in the Manhattan plot. The modified *p*-values are displayed on the *y*-axis in negative log_10_ scale. Every circle represents one phrase and is proportional to the term size, i.e., larger terms have larger circles. The Supplementary Material includes an interactive version of this plot (Manhattan_plot_GSEA_Gost.html). Hovering over the circle in the interactive plot will display the appropriate information. If the −log_10_ (*p*-values) exceed 16, they are capped at 16. This adjusts the *y*-axis scale to keep Manhattan plots from different queries similar, and it is also intuitive because statistically, *p*-values less than that can all be summarized as highly significant.

Cluster 12 contains 199 genes, which a functional analysis implemented with the enrichGO function of R library clusterProfiler for the Gene Ontology (GO) Enrichment Analysis (Yu, 2012; Yu et al., 2012) finds to have the molecular functions shown in barplot of Figure 9 and the ontologies of the cellular compartments as in Figure 10. The list of the gene names of Cluster 12, as well as the summary of enrichGO and of gost function of the R library gprofiler2 (Raudvere et al., 2019; Kolberg et al., 2020; Raudvere et al., 2023) are available in the Supplementary Material. To give a more complete view of the results of the gene set enrichment analysis of Cluster 12, we show in Figure 11 the Manhattan-plots of the gene set enrichment analysis, of which we also give an interactive version in the Supplementary Material.

Of interest is a result shown in Figure 9, namely, the presence in Cluster 12 of genes co-involved in the molecular processes of “SMAD binding”. Smad proteins, are central mediators of the signal transduction of TGF-*β* family members were identified in the dataset analysed. A Cross-talk between TGF-*β*/Smad pathway and Wnt/*β*-catenin pathway in pathological scar formation has been described suggesting a complicated interaction between the two signal pathways in pathological scar formation (both synergy and antagonism) (Sun et al., 2015). More recently TGF-*β*/SMAD, Hippo/YAP/TAZ, and Wnt/*β*-catenin signalling pathways, major inducers of transcriptional reprogramming, were shown to converge at several levels and were all required for a proliferative-to-invasive phenotype switch in melanoma development (Lüönd et al., 2021). We already described in a previous study the involvement of Wnt/*β*-catenin signalling pathway in the tumour suppressor effect driven by PTPRG in CML (Tomasello et al., 2020) and the current data reporting the involvement of SMAD pathway is in line with a complex cellular reprogramming induced by PTPRG expression whose key role in the haematopoietic differentiation program was already described (Sorio et al., 1997). This complex reprogramming is supported by the large number pathways involved in DNA binding/transcription reported on Cluster 12 GSEA. In particular, in our previous study (Lombardi et al., 2022), we validated the SMAD1 gene. Specifically, qRT-PCR was used to assess gene mRNA levels, and the relative fold changes were calculated between K562 expressing PTPRG and the untreated control group (control and D1028A). The endogenous control was GAPDH. We found that the fold change of SMAD1 is markedly greater in the case of the control [see Figure 3 of Lombardi et al. (2022)].

### 5.1 Comparison with spectral clustering

**FIGURE 12:**
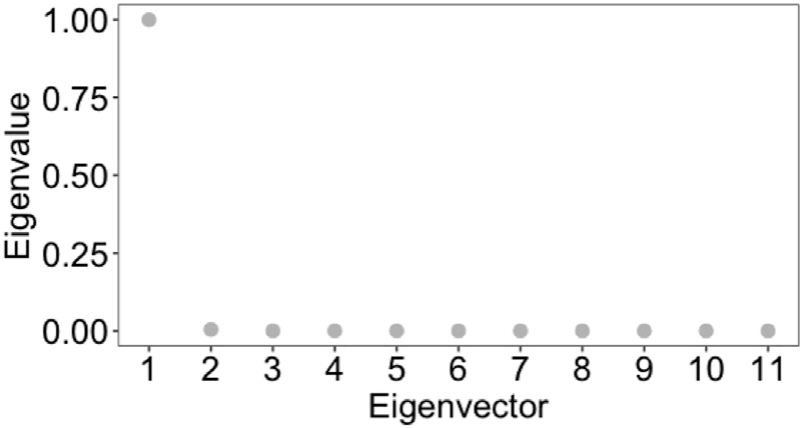
Eigengap heuristic: the optimal number of clusters, *k*, that maximises the eigengap (difference between consecutive eigenvalues of the Laplacian matrix of the graph). The optimal number of clusters is that k such that *λ*_*k*+1_ is reasonably large but all other eigenvalues, *λ*_1_, *…* , *λ*_*k*_, are very small. The closer the eigenvectors of the ideal case are, and hence the better spectral clustering performs, the wider this eigengap is.

We compared the results of the clustering by radial coordinate in hyperbolic space with the spectral clustering method. This method is widely used to identify communities of nodes in a network by examining the edges that connect them, i.e., taking as an input the weighted adjacency matrix of the graph. It is a well-established method with theoretical foundations in graph theory (we refer the reader to JingMao and YanXia (2015); von Luxburg (2007) for a review and a tutorial on this popular spectral clustering methods). Processing directly the weighted adjacency matrix of the graph, that is the same input as our embedding procedure, we consider spectral clustering to be the most appropriate method to deal with, compared to clustering methods based on graph centrality measures, or on statistical correlation measures between nodes, who do not into account directly distance measures between nodes. Before applying spectral clustering, we estimated the optimal number of clusters with eigengap heuristics [appropriate procedure for estimating the number of clusters for spectral clustering methods (von Luxburg, 2007)], obtaining that the optimal number of clusters is 2 (see Figure 12). Cluster 1 contains 1,731 nodes and cluster 2 contains 349 nodes. Using the R script Spectral_clustering.R to implement spectral clustering - available in GitLab repository, we found that the within cluster sum of squares by cluster is 1.1079409 and 0.2348562, whereas the between sum of squares is 0.6502382. As a consequence, we conclude that the results of the spectral clustering are not reliable. This result highlights how taking into account the latent geometry of the network and with it the clustering according to the spatial co-ordinate of the nodes/points of the network resulted in a much better quality of clustering, compared to a clustering which, as in our approach, processes the weighted adjacency matrix, but does not consider the latent geometry of the network expressed by the position of the points in the optimal embedding space and the distance defined by the metric in this space.

That various clustering methods are not appropriate for graphs with geometry has also been pointed out by Avrachenkov et al. (2021) that states that while it has been demonstrated that spectral clustering is consistent in some geometric graphs, a cut-based technique (such as spectral clustering) can also be significantly hindered by the geometric structure. It is possible to divide space into regions in such a way that there is relatively little interaction between nodes in two different regions. Therefore, the Fiedler vector of a geometric graph may only be linked to a geometric arrangement and contain no information regarding the labelling of the latent community. Furthermore, because the regions of space can include a balanced number of nodes, the widely used regularisation strategy (Zhang and Rohe, 2018), which seeks to penalise small size communities in order to bring back the vector associated with the community structure in the second position, would not function in geometric graphs.

## 6 Conclusion

In this study, we modelled the transcriptome network of the of Chronic Myeloid Laeukemia K562 cells overexpressing the tumour suppressor gene PTPRG, as a physical system of springs and then deduced the spring constant from topological properties of the nodes, such as total degree. To represent the network, we considered the dissimilarity matrix consisting of the values of the spring’s elastic constant, which in our model quantifies the efficiency of information transmission between nodes. Through network embedding procedures that processed the dissimilarity matrix to derive the coordinates of the nodes in a metric os pact we determined the optimal latent geometry of the network is hyperbolic. This important information made it possible to proceed with the classification of nodes according to radial co-ordinates (which is the geometric equivalent of the ‘physical’ concept of node popularity) and to identify a set of candidate driver genes for network dynamics.

This methodology aimed at analysing a network without ignoring the existence of its metric space with a geometry other than the Euclidean one usually imposed or taken for granted, shows how latent geometry can determine a classification of nodes according to their relevance in the network’s evolutionary processes, ultimately its dynamics. In the particular case study presented here we obtained that the network has hyperbolic latent geometry, and based on this we proceeded to utilise the concept that in this type of geometry the radial coordinate is a fundamental variable for clustering nodes. Geometries other than hyperbolic are characterised by other spatial variables that can be considered discriminating for the purpose of identifying driver nodes of the dynamics. What is presented in the paper, besides being a concrete result on a specific case study, is also a proposal for a method of analysing a network in order to reveal information about the dynamics of the network itself.

## Data Availability

The original contributions presented in the study are included in the article/Supplementary Material, further inquiries can be directed to the corresponding author.
